# Patients with Non-Specific Complaints in Emergency Departments: A Growing Patient Safety Concern in an Aging Population with Multimorbidity

**DOI:** 10.3390/healthcare12202014

**Published:** 2024-10-10

**Authors:** Ninna Meier, Kirstine Zinck Pedersen, Linda Camilla Andresen, Ove Andersen

**Affiliations:** 1 Department of Sociology and Social Work, Aalborg University, 9220 Aalborg, Denmark; 2 Department of Organization, Copenhagen Business School, 2000 Frederiksberg, Denmark; kzp.ioa@cbs.dk; 3 Department of Clinical Research, Copenhagen University Hospital Amager and Hvidovre, 2650 Hvidovre, Denmark; linda.camilla.andresen@regionh.dk (L.C.A.); ove.andersen@regionh.dk (O.A.); 4 Emergency Department, Copenhagen University Hospital Amager and Hvidovre, 2650 Hvidovre, Denmark

**Keywords:** patient safety, non-specific complaints, diagnosis, emergency departments

## Abstract

In this opinion, we offer a new perspective on the important and persistent problem of diagnostic errors for patients with non-specific complaints (NSCs). As an increasing number of complex patients present clinicians with challenging diagnostic work in the time-pressured and high-volume contexts of EDs, we need to improve how clinicians and healthcare organizations can understand and perform safe diagnostics for patients with NSCs. The combination of a growing number of patients with NSCs and the ways in which clinicians use the categories ‘non-specific complaints’ and ‘non-specific diagnosis’ in diagnostic work in emergency departments presents a growing patient safety concern especially for older patients with multimorbidity that require the integration of clinical and organizational research. We argue why the growing numbers of patients with NSCs and clinicians’ use of these categories have implications for patient safety both within and beyond the acute care context. We end by pointing to the importance of an interdisciplinary patient safety research agenda, ideally followed by the development of targeted usable protocols for older multimorbid patients with non-specific complaints.

## 1. Introduction

This opinion proposes a new way of understanding, researching, and improving patient safety for patients with non-specific complaints (NSCs). NSCs refer to conditions, such as fatigue, that can occur in effectively any known illness and therefore are not specific for any diagnosis but are especially common when patients are older, suffer from multimorbidity, and when they present with atypical and interacting symptoms. The core of the problem, we believe, arises from several interconnected elements: firstly, the rise of patients with non-specific overlapping complaints, complex and often atypical clinical presentations, and a heightened risk of adverse drug reactions; secondly, the traditional diagnostic system’s deficiencies in adequately categorizing and identifying risks to patient safety across different diagnostic codes, especially during the initial triage and treatment processes in emergency departments (EDs); and thirdly, the insufficient recognition of EDs as specific organizational contexts that perform an important function for early interventions in a patient’s pathway due to their role as a gateway into specialized hospital treatment and their connection to primary care and the larger healthcare system.

## 2. Patients with Non-Specific Complaints

Diagnostic work—the process of determining the nature of a disease and distinguishing it from other possible conditions—ideally involves assessing all the relevant information on distinct complaints, symptoms and their severity, causations, potential disease development, treatment options, and predictions. Non-specific complaints such as dizziness, headache, and fatigue can have many causes, they often overlap, and they can be signs of an illness that presents atypically in multimorbid patients making diagnostic work time-consuming and difficult. While the current studies use different definitions and applications of non-specific complaints in their analysis, in practice, these complaints are among the most frequent symptoms that patients present with in EDs (20% in adult populations and up to 20% of older patients with a risk of further health deterioration) [1] and a considerable number of these patients receive a non-specific diagnosis at discharge from EDs [2]. Thus, because the category ‘non-specific diagnoses’ may seemingly offer a practical solution to this challenge, patients with non-specific complaints have an increased risk of being (only) categorized with a non-specific diagnosis as the outcome of the diagnostic work.

However, when populations live longer with multiple and/or chronic diseases, both coexisting diseases (multimorbidity) or as occurrences of distinct additional diseases (comorbidity) [3], this practical solution can ‘hide’ the significant potential for improvement [4]. In acute care, patients with NSCs are triaged as less urgent than patients with disease-specific complaints, and they have a higher mortality, longer in-hospital stays, and experience more frequent readmissions [5]. From here, the findings from prior research diverge. Some studies found ‘that NSC is significantly associated with lower utilization of ED diagnostic resources’ (9% less) [1], with regard to, e.g., material, laboratory, and radiology resources, but also associated with lengthened hospitals stays [6] and resources spent on direct patient contact. Others have found the opposite when it comes to diagnostic tests and procedures in cohorts of elderly patients with weakness and fatigue [7]. This calls for a better understanding of how, where, and when, the treatment of patients with NSCs differs from other patient categories in order to understand the categorizations which impact on patient outcome, quality of care, and costs [8].

Lastly, patients with NSCs as a group are recognized as an emerging and widespread driver of emergency department crowding [9], affecting the quality of diagnoses and accentuating the overall impact on patient safety across the entire intake of patients beyond the sheer volume of patients with NSCs. At the same time, the International Classification of Diseases (ICD) codes are more difficultly posed for these patients due to the non-specificity of their complaints. Clinicians can then either spend more resources on diagnostic work for these patients, which requires all patients to wait longer in the ED before discharge, compromise flow, and potentially exacerbate ED crowding, or they can discharge the patient admitted with NSCs with a non-specific diagnosis after a triage assessment, which can increase the risk of readmission for patients with multiple comorbidities and functional impairment [10].

## 3. The Use of Non-Specific Categories

Several factors may increase clinicians’ use of non-specific categories in EDs and for older patients, as symptoms of frailty and aging interact in complex ways with diseases and mental status. Some factors are clinical, while others arise as a consequence of the organization of clinical work, founded on a system of diagnostic coding. However, regardless of the reasons, the use of non-specific categories can pose a threat to patient safety improvements for older multimorbid patients in the ED and beyond. Non-specific complaints and non-specified diagnoses can be understood as residual categories, used for items that cannot solely be assigned to one of the other categories in a classification system, for instance when ‘a medical condition does not fit one of them’ [11] (p. 274). Such categories are important for the flexibility and usability of any classification system in general.

In the clinical practice of EDs, the non-specificity of this residual category (‘non-specific’) allows clinicians to maintain flow and effectively perform the preliminary ‘sorting’ of patients. However, residual categories aid the usability of the general classification system only as long as the use of the category does not effectively supersede the use of specific categories and as long as the use of such a category does not hinder important insights, which we suggest is now the case for the overall population of patients with NSCs. If clinicians’ diagnostic work (only) results in the registration of ‘non-specific complaints’ and/or ‘non-specific diagnosis’, our ability to systematically investigate how diagnostic errors arise when patients present with overlapping, general complaints, or atypical presentations of disease is hampered, and the implications may spill-over into work performed in other organizational contexts such as general practice or nursing homes in part due to the increasingly digitalized communication accompanying patients’ transitions between healthcare sectors.

## 4. EDs as Sites of Early Interventions

EDs are often the first entrance into the specialized healthcare system and thus an important junction for early interventions. The admission to ED marks a change in a patient’s subjective perception of being well to being potentially ill, aided by a concerned referral from primary care caused by acute manifestations of (severe enough) illness. However, while EDs serve each individual patient, they also play a vital part in maintaining the patient flow and bed capacity in the larger healthcare system. As clinicians in EDs provide initial diagnoses and treatments for patients, their role in the healthcare system is to ‘sort out’ which patients need hospitalization and which patients can be discharged home to the responsibility of the primary care sector’s providers. So, while the clinical decisions made here are essential for individual patients, organizations, and the coherence of the overall healthcare system, these decisions are often made under challenging conditions. According to a recent systematic review [2], 5.7% of ED patients are discharged with at least one diagnostic error, 2% with a potential harmful diagnostic error, and 0.3% with a diagnostic error that causes serious injury. These numbers are comparable with other sectors of the healthcare system, including specialized care, but the difference lies in the total volume, i.e., the high number of patients that ‘transit’ through EDs.

EDs are characterized as flow cultures, where physicians and nurses have limited time to exercise their judgement and reflect on their clinical decision [12] This is exacerbated by organizational factors typical for EDs such as off-hours presentations, urgent handoffs, staffing shortages, and crowding. Together, these conditions increase the risks to patient safety arising from diagnostic errors from missed, delayed, or wrong diagnoses, or insufficient qualification of any potential risk to the patient later in the flow; for instance, if a patient with NSCs is discharged with only ‘non-specific diagnosis’, vital information can be lost in the transition to the patient’s own home under the responsibility of primary care providers.

Diagnoses, specific as well as the residual (non-specific), are part of a shared classification system, the primary system being the ICD-11. Classification systems of diagnoses were initially conceptualized as a tool for clinicians to support knowledge sharing and clinical decision-making through diagnostic classification groups that are mutually exclusive [13]. Classification criteria consist of standardized terminology and are often used in clinical research or descriptions of syndromes to create well-defined and homogenous cohorts with shared features. Classification criteria and diagnostic criteria can be understood as representing ‘two ends of a continuum’ [14] where the distance between them depends on factors such as expected diagnostic prevalence in a given cohort, the clinical knowledge at the site, the organization of work, contextual collaboration with other medical specialties, and discharge practices, etc.

Classification systems such as the ICD-11 are part of a larger infrastructure for clinical knowledge that connects diagnostic work to a range of related activities, e.g., efforts to monitor quality and patient safety issues, economic reimbursement programs, public health interventions, and clinical research [15]. Through this connectedness with other key elements of the healthcare system, disease classification systems and diagnostic codes may fade into the background as taken-for-granted aspects of clinical work that mainly call attention to themselves only when they break down or do not work in practice [16], as when patients present with multiple, overlapping, non-specific complaints.

The diagnostic system provides infrastructure for clinicians’ diagnostic work and collaboration within specialized healthcare systems, but patients with NSCs are not only handled in specialized care; this patient group is mostly handled in primary care where the diagnostic system is not the main infrastructure for knowledge, communication, and collaboration. The lack of precision that comes with the use of a ‘non-specific diagnosis’ travels into primary care with the vulnerable, multimorbid patient suffering from the often several, overlapping, non-specific complaints and may onset a futile cycle of readmissions and (re)discharges. Thus, the non-specific needs to be qualified, not by adding yet another number of diagnostic codes for registrations, but by aligning the system and our way of diagnostic work to a new demographic reality where multiple chronic diseases develop concurrently with overlapping or even similar risk patterns.

## 5. Discussion

Currently, we have no good strategies for researching or improving the potential consequences of the increased use of residual categories in EDs on patient safety. In the last two decades, patient safety research has adopted an approach of isolating errors and improving safety in specific procedures, often standards or technology, based upon an analysis of what went wrong in single cases. Existing methods to study and improve patient safety have thereby focused on dealing with tangible, easily identifiable adverse events and their causes, rather than complex diagnostic processes [17]. By focusing on minimizing and preventing single, codifiable errors through retrospective analyses, insufficient attention is given to how clinical decision-making is practiced in and between different organizational and clinical contexts. Retrospective analysis of what went wrong often results in the introduction of various types of standardization in the form of variance-reducing procedures or technological fixes to prevent similar errors in the future. However, such interventions do not consider that healthcare organizations are open and complex systems in which the conditions that lead to diagnostic errors or unsafe situations are difficult to isolate and rarely precisely identical. As improvements based on this approach have also been found to be slow and spotty, other approaches that take the point of departure in the situated and uncertain nature of clinical decision-making and investigate aspects such as ‘safety culture’ and ‘safety dispositions’ need to gain more traction [18,19,20].

## 6. Implications for Policy, Research, and Practice

While the use of non-specific categories is growing due to increased complexity in disease manifestation in an aging demographic, the systemic demand for accuracy in diagnostics, and maybe more importantly, the importance of documentation hereof for healthcare projections, policies, and management, has gained importance and diagnostic data is increasingly used outside the daily clinical work as a means of evaluation, administration, and management of healthcare organizations. Unfortunately, the combination of increasing numbers of patients with NSCs and the ways in which the residual category of NSCs is used in practice in diagnostic work, and the consequences of this for patient safety, is currently under-researched relative to its importance for clinical practice and research. This lack of a solid scientific foundation is to the disadvantage of patients, clinicians, organizational improvement, and research efforts, as well as the development of patient safety policies. How can we remedy this?

Diagnostic work and clinical decision-making are practices that involve an element of judgement, require complex professional expertise, and take time and practice to learn. During the diagnostic process, the clinician must decide what to pay attention to, what to ascribe significance to, and what to eliminate as ‘not relevant’ in the context, e.g., due to interference or perceived non-importance. An example is the need to identify those elderly people who are at high risk of falling because of poor balance. Here, the diagnostic boxes will demand a cause of the fall risk, e.g., fluid imbalance, delirium, side effect from drugs, or micro-fractures. However, for an older patient with multimorbidity, all of these could in isolation or in combination cause an increased risk of falling. In such cases, the main information needed for a clinician caring for this patient is the risk of falling as a safety concern (and subsequently, its causes) if the clinician is to prevent further harm in the present. Thus, the most important information for a clinician caring for this patient is the presentation of symptoms that require attention now and to rule out more severe underlying diseases, including the interpretation of what might be causing these symptoms. We propose that understanding, defining, and performing the dual assessment centered on risk identification and increased specificity in diagnostic work is perhaps the most important task, both on an individual and organizational level, if we are to improve patient safety for patient with NSCs, and for this we need an interdisciplinary approach in several steps.

The first step is to systematically investigate where, when, how, and why risks to patient safety arise in diagnostic work for patients with NSCs in clinical practice. It is important that such studies are carried out with a combination of methods that allow researchers to capture the practices, times, and places where improvements are most needed and can have beneficial implications across healthcare systems. One such place is in acute care, specifically in EDs that are tasked with admission, discharges, and referrals across disciplinary, professional, organizational, and geographical boundaries in the healthcare system.

Based on such interdisciplinary research, the second step is to develop practically useful improvements to patient safety for patients with NSCs through organizational interventions and healthcare policies based on systematic research. Here, interdisciplinary methods that investigate and reflect the conditions for diagnostic work in acute care are necessary if we are to improve the treatment and safety outcomes for this patient group. When investigating and improving patient safety for patients with NSCs, it is necessary to continuously take the complex nature of the issue into account. Therefore, interventions should not be introduced without a thorough understanding and assessment of the conditions that have led to the use of residual categories and an analysis of possible unintended consequences of intervening in one part of the healthcare system for other parts. Some pertinent tensions and dilemmas must be considered.

First, interventions must acknowledge that a problem for patient safety lies in a combination of increasing numbers of complex patients and the increasing use of residual categories in diagnostic work as a way to handle the practical impact of these patients in EDs.

Thus, the use of residual categories is an answer to at least two different but interrelated problems: a rising amount of complex and often multimorbid patients with diffuse symptoms, and an often-overstretched acute care setting where these categories can be used as organizational tools for speeding up diagnostic processes in times when the ED is crowded [9]. Thus, to develop appropriate interventions, a first research objective is to gain a better overview of the use of residual categories and to understand the complex reasons behind the increasing numbers.

Second, the pressure for quick decisions in the acute care setting and the need for thorough assessments when dealing with NSCs create an inherent dilemma. Therefore, interventions that seek to better the conditions for clinical decision-making—whether by, e.g., increasing time for the diagnostic process for complex patients, increasing interdisciplinary decision-making and teamwork, adding decision-making support systems (e.g., AI), or introducing new triage or risk profiling systems—must take into account the ever-present possibility that these new interventions can hamper the flow and speed of the diagnostic process in the ED, with consequences for equally complex and more standard patients with acute care needs. This calls for caution and thorough analysis when introducing new interventions, and it calls for pilot tests closely followed by interdisciplinary teams of researchers to access equally clinical and organizational effects of interventions before decisions are made about scaling and disseminating these.

Third, the lack of specificity in the ‘non-specific diagnosis’ category travels with the patient whether into primary care settings, specialized hospital settings, or the patient’s home or aged care facility. Interventions must consider that when patients are discharged or transferred without sufficient diagnoses, this might impede or delay specialized treatment, with negative effects for patient safety and for the overall efficiency and flow of patient pathways in the healthcare system. Moreover, the majority of patients spend most of their time at home, cared for by staff from primary sector organizations, where insufficient diagnoses might result in inappropriate treatment strategies or insecurities for equally primary healthcare staff, patients, and relatives. Thus, while the use of residual categories might help optimize processes in the ED when time and resources are scarce, they have unintended consequences for the rest of the healthcare system. Therefore, any intervention must consider the entire patient trajectory, the various transitions between healthcare systems, and the possible compensatory consequences that optimizing one part of the system might have on other parts. Because the mentioned dilemmas are thoroughly interconnected and not readily dissolvable, we propose that improvement efforts and interventions include elements focusing on strengthening the organizational coherence in patient pathways in EDs and beyond. This might include joint activities designed to develop inter-organizational collaboration and feedback learning to all the involved parts of the healthcare organization. For instance, continuous improvement processes where concrete cases of non-specific complaints or diagnoses are used as learning opportunities to refine diagnostic pathways and improve care processes throughout the healthcare systems.

As a third step, it will become important to improve the evidence base and systematization of diagnostic pathways and treatment protocols for patients with NSCs, and to evaluate the need for making more macro-level national and international changes in diagnostic coding systems and healthcare governance structures. When developing evidence-based and targeted protocols, these must take the heterogenic manifestations of these conditions, their clustering and distributions, and risk identification and profile into account. Importantly, such protocols must be based on the identification and acknowledgement of patient safety concerns that are involved in the diagnosis of patients with non-specific complaints along all the steps of the patient pathway and outline the ways in which clinicians need to be supported to address such issues in practice, to avoid harm. Moreover, the increasing numbers of multimorbidity and older persons with multiple chronic diseases necessitate a critical look at the diagnostic criteria [10], the specific and the non-specific, and their performative effects in the clinic and beyond. This is of course in full recognition of the still unknown etiology and pathogenesis behind the co-dependent development of diseases in an aging body, often complicated by random or dependent intercurrent acute illnesses, and polypharmacy. Lastly, healthcare governance is a major factor in handling interdisciplinary and cross-cutting patient safety problems. We know that integration, collaboration, and coordination between health units and systems can be hampered by decentralized budgetary responsibility and competing performance or production measures [21,22]. While these governance remnants from the New Public Management era are today massively criticized, and new more collaborative models to solve more complex healthcare problems are suggested based on the ideals of collaboration, network, and partnership [23,24], healthcare governance structures are still often reproducing the tendency to optimize within units rather than between them [25]. A dominant countertrend here is the expansion of clinical pathways as an organizational tool to increase coordination in healthcare [26,27]. However, as pathways are pre-dominantly disease-specific, they might well increase coordination for standard patients but can have opposite effects for patients that do not fit the boxes due to for instance co- or multimorbidity or missing diagnoses. In the quest to optimize patient safety for patients with NSCs, governance structures must therefore also be addressed, for instance to support incentives for extending responsibility for patients beyond discharge and strengthen the collaboration and coordination of non-standard patients’ diagnostic and treatment processes through alternative clinical pathways or treatment guarantees.

Multimorbidity will hopefully in the foreseeable future be the focus of new evidence-based treatment, clinical pathways, and care protocols, but any successful outcome of their implementation will rely on knowledge of how to identify and define multimorbidity from other diagnoses, and how multimorbid patients can benefit in terms of safety and wellbeing from another type of approach to their complaints, including risk profiling. Another main challenge here is that multimorbid patients, and especially multimorbid patients with polypharmacy, are still often excluded from clinical research trials and omitted from epidemiological conclusions due to their apparent heterogeneity in diagnostics. Qualifying the use of classification criteria and refining them to distinguish symptoms in populations with multimorbidity and functional impairments are necessary steps in the ongoing evolution of the ICD before developing new interventions [28]. Additionally, it must be recognized that new classification criteria targeting multimorbid patients are insufficient solutions to the patient safety challenges that patients with NSCs face unless they are supplemented with a focus on how clinical, organizational, and policy elements interact in both research and improvement practices.

## 7. Conclusions

Healthcare practices are changing under the impact of medical and societal innovations, demographic and organizational changes, and political programs. Our basic understanding of what constitutes a diagnostic error for complex multimorbid patients and how to measure and counteract its consequences should change in parallel, incorporating the abovementioned factors. In this opinion, we point to the importance of more conceptual clarity and improved use of the category ‘non-specific’ in diagnostic processes. We also argue in favor of a more in-depth interdisciplinary approach to where, when, how, and why risks to patient safety arise in diagnostic work, to address the growing challenges in this area. By reconsidering the system and uses of diagnostic coding as well as the challenges of coordination and collaboration in the current healthcare organization and governance structures, new potentials for improving patient safety can be explored across healthcare boundaries with EDs as a central starting point for our investigations.

## Data Availability

No new data were created or analyzed in this study.
